# ‘First, do no harm’: are disability assessments associated with adverse trends in mental health? A longitudinal ecological study

**DOI:** 10.1136/jech-2015-206209

**Published:** 2015-11-16

**Authors:** B Barr, D Taylor-Robinson, D Stuckler, R Loopstra, A Reeves, M Whitehead

**Affiliations:** 1 University of Liverpool, Liverpool, UK; 2 Oxford University, Oxford, UK

**Keywords:** DISABILITY, Health inequalities, POLICY

## Abstract

**Background:**

In England between 2010 and 2013, just over one million recipients of the main out-of-work disability benefit had their eligibility reassessed using a new functional checklist—the Work Capability Assessment. Doctors and disability rights organisations have raised concerns that this has had an adverse effect on the mental health of claimants, but there are no population level studies exploring the health effects of this or similar policies.

**Method:**

We used multivariable regression to investigate whether variation in the trend in reassessments in each of 149 local authorities in England was associated with differences in local trends in suicides, self-reported mental health problems and antidepressant prescribing rates, while adjusting for baseline conditions and trends in other factors known to influence mental ill-health.

**Results:**

Each additional 10 000 people reassessed in each area was associated with an additional 6 suicides (95% CI 2 to 9), 2700 cases of reported mental health problems (95% CI 548 to 4840), and the prescribing of an additional 7020 antidepressant items (95% CI 3930 to 10100). The reassessment process was associated with the greatest increases in these adverse mental health outcomes in the most deprived areas of the country, widening health inequalities.

**Conclusions:**

The programme of reassessing people on disability benefits using the Work Capability Assessment was independently associated with an increase in suicides, self-reported mental health problems and antidepressant prescribing. This policy may have had serious adverse consequences for mental health in England, which could outweigh any benefits that arise from moving people off disability benefits.

## Background

Several measures indicate that mental health in the UK has deteriorated in recent years, with suicides reaching a 13-year high in 2013.^1–3^ We have previously shown that an upturn in suicides was associated with the 2008–2010 recession,^2^ however these trends have continued to worsen even after the economy recovered.^3^ Since 2010 over a million claimants of the main out-of-work disability benefit in the UK had their eligibility reassessed using a new functional checklist—the Work Capability Assessment (WCA).^4^ Doctors and Disability groups have raised concerns that this reassessment process has had a negative effect on the mental health of their patients.^5–7^

The provision of cash benefits to people who are unable to work because of disability is an essential component of health and welfare systems that aim to promote the social inclusion of people with disabilities.^8^ Over recent years many countries, including the UK, the Netherlands and Australia, have introduced more stringent functional assessment checklists to reduce the growing number of people receiving disability benefits.^9^ ^10^ While in most countries these more stringent criteria have only been applied to new benefit claimants, the UK and the Netherlands have gone further—reassessing their entire caseloads.^8^ In the UK this process started in 2010 when the government initiated a programme to reassess all existing claimants of out-of-work disability benefits using the WCA. Following reassessment the claimants were either moved off disability benefits, if found to be fit for work, or otherwise were transferred to a new disability benefit scheme called Employment Support Allowance.

The WCA has been the subject of a great deal of controversy. Nearly 40% of those who have appealed against the initial assessment decision have had this decision overturned,^11^ and five independent reviews have raised concerns about the fairness and effectiveness of the process. In particular the reviews indicated that the process was impersonal and mechanistic and did not adequately capture the impact of many chronic health conditions.^12^ The government has however accepted many of the recommendations of these reviews and changed the WCA over time. Many of these changes have particularly focused on the assessment of mental health problems, including adjustments to the mental, intellectual and cognitive descriptors, additional training of decisionsmakers and assessors and the appointment of Mental Function Champions.^13^

Several anecdotal reports and surveys of doctors describe individuals experiencing a deterioration in their mental health and even suicides following their WCA.^5^ ^6^ ^14^ ^15^ Psychiatrists in one survey reported that some patients had experienced an increased frequency of psychiatric appointments, medication usage and self-harm following their WCA.^14^ These anecdotal reports, however, provide limited scientific evidence for the mental health effects of the WCA.

Both the assessment and appeals process itself, which is reported to be stressful, and the financial hardship that occurs when people are denied disability benefits, could result in negative health effects. There is good evidence that loss of income, particularly for people already on low incomes, increases risk of common mental health problems.^16^ People undergoing a WCA are likely to be particularly vulnerable to the adverse mental health consequences of this policy because a very high proportion have a pre-existing mental health problem.^17^ A previous study in Norway reported an increase in mental health symptoms leading up to the time when new applicants began receiving disability benefits,^18^ however this study did not investigate how mental health changes when current recipients of disability benefits have their eligibility reassessed.

Understanding the benefits and harms of these eligibility assessments is of international importance both for the health professionals who implement the assessments and for policymakers who need to decide on the most effective approaches. While the potential effects on employment prospects are debateable,^19–21^ to our knowledge no studies have assessed the impact of the disability assessment process on the mental health of the recipients. We took advantage of the variation across local authority areas in the rate at which this reassessment process took place, to investigate whether this policy was associated with an increase in three mental health outcomes collected in different data sets—suicides, self-reported mental health problems and antidepressant usage.

## Methods

### Setting

We used aggregate routine population and survey data for 149 upper tier local authorities in England between 2004 and 2013. (The City of London, Rutland and the Isles of Scilly were excluded due to their small population size). Analysis was restricted to England as comparable data were not available for Scotland and Wales.

### Data sources and measures

We used three outcome variables in our analysis; suicides, antidepressant prescriptions and self-reported mental health problems. Age-adjusted mortality rates from suicide and injury of undetermined cause in the working age population (18–64) were obtained for each local authority between 2004 and 2013 from the Office for National Statistics. We calculated quarterly antidepressant-prescribing rates per 100 000 population, for each local authority area from 2010 (the earliest available year) to 2013 using data on antidepressant items prescribed by each general practitioner practice aggregated up to the local authority level.^22^ We estimated quarterly prevalence rates of self-reported mental health problems per 100 000 working age population (18–64 years old) for each local authority between 2004 and 2013 using data from the Quarterly Labour Force Survey (QLFS) adjusted for response bias using survey weights supplied by the Office for National Statistics.^23^ Details of the survey questions used are given in online supplementary appendix 1.

Our main exposure variable, the reassessment rate, was the cumulative proportion of the working age population in each local authority area that had received any outcome from a WCA as part of the reassessment process, by the end of each quarter, expressed as a rate per 100 000 population (ie, the cumulative incidence of reassessment).^11^ We used the cumulative proportion of the population exposed as our main measure in order to investigate the accumulated effects of the policy on mental health outcomes. In additional analysis we also used the quarterly incidence of reassessment, calculated as the number of outcomes received in each local authority area during each quarter as a proportion of the population.

We also included measures of area deprivation using the Indices of Multiple Deprivation (IMD 2010)^24^ and controlled for differences in economic trends between areas using the annual regional workplace-based gross value added (GVA) per capita (the regional equivalent to gross domestic product), the quarterly unemployment rate (based on unemployment benefit claimant data) and the annual median wages of residents in each local authority area.^25^ ^26^ To adjust for any local effects of changes in local authority spending we additionally controlled for annual trends in public expenditure by local authorities.^27^

### Analysis

To explore the data visually, we used added variable plots^28^ to described the association between the proportion of the population reassessed in each local authority area between 2010 and 2013 and the change in each of our outcomes (suicides, self-reported mental health problems and antidepressant prescribing) between these years, while controlling for baseline area deprivation. Owing to the small numbers in each local authority in each year we pooled data over 2 years and calculated the change as the difference in each outcome between 2009–2010 and 2012–2013.

We then used linear fixed effects multivariable regression models to formally test this association while further adjusting for other potential confounding factors.^28^ As suicide mortality data were only available annually, annual panel data were used for this outcome, while for all other outcomes quarterly panel data were used. By including a fixed effect for each local authority, we effectively control for all baseline differences between local authority areas, including the baseline prevalence of benefit receipt, so that our models assessed the association between the trend in the reassessment rate and the trend in outcomes within each local authority.^29^ As the trends in the reassessment process were correlated with economic trends (see online supplementary appendix 5) and these could influence mental health outcomes, we further controlled for trends in GVA per capita, median wages and unemployment rates. As there were two changes to the health module of the QLFS questionnaire during this time in 2010 quarter 1 and 2013 quarter 1, we included dummy variables in our models to account for any discontinuities in the data at these time points. (see online supplementary appendix 1 for details).

We include data in these models from 2004 in order to account for pre-existing trends in our mental health outcomes. Bias could result if associations between the reassessment policy and mental health outcomes were actually due to differential pre-existing trends, that started before the onset of the policy.^30^ Therefore, to adjust for these pre-existing trends we included trend terms in all models and allowed these trends to vary in the period prior to the economic crisis (2004–2006) and in the period during and after the economic crisis (2007–2013). As the reassessment process followed differential regional trends with the North East, North West, and more deprived areas affected to a greater extent (see online supplementary appendix 5) we controlled for this by including separate time trends for each government office region in England and each quintile of area deprivation (IMD). In a sensitivity analysis we estimated models with simpler time trend assumptions including models with just a national level linear time trend and models just including data during the period in which the policy was implemented (2010–2013; see online supplementary appendix 4.)

To investigate the specificity of our results we repeated the analysis using outcomes we would not expect to be influenced by the reassessment policy, but that could be affected by unobserved confounding factors. These included mental health problems and suicides in people over the retirement age of 65, heart conditions in the working age population and items of cardiovascular drugs prescribed per 100 000 population. We further investigated whether trends in adverse mental health outcomes were a response to the reassessment rate by estimating additional models including the lagged quarterly incidence of reassessment (ie, the proportion of the population receiving an outcome from the reassessment process in the previous quarter), rather than the cumulative incidence of reassessment (see online supplementary appendix 4). We used robust clustered SEs in all models to account for the longitudinal nature of the data and weighted the analysis by local authority population.

## Results

**Figure 1:**
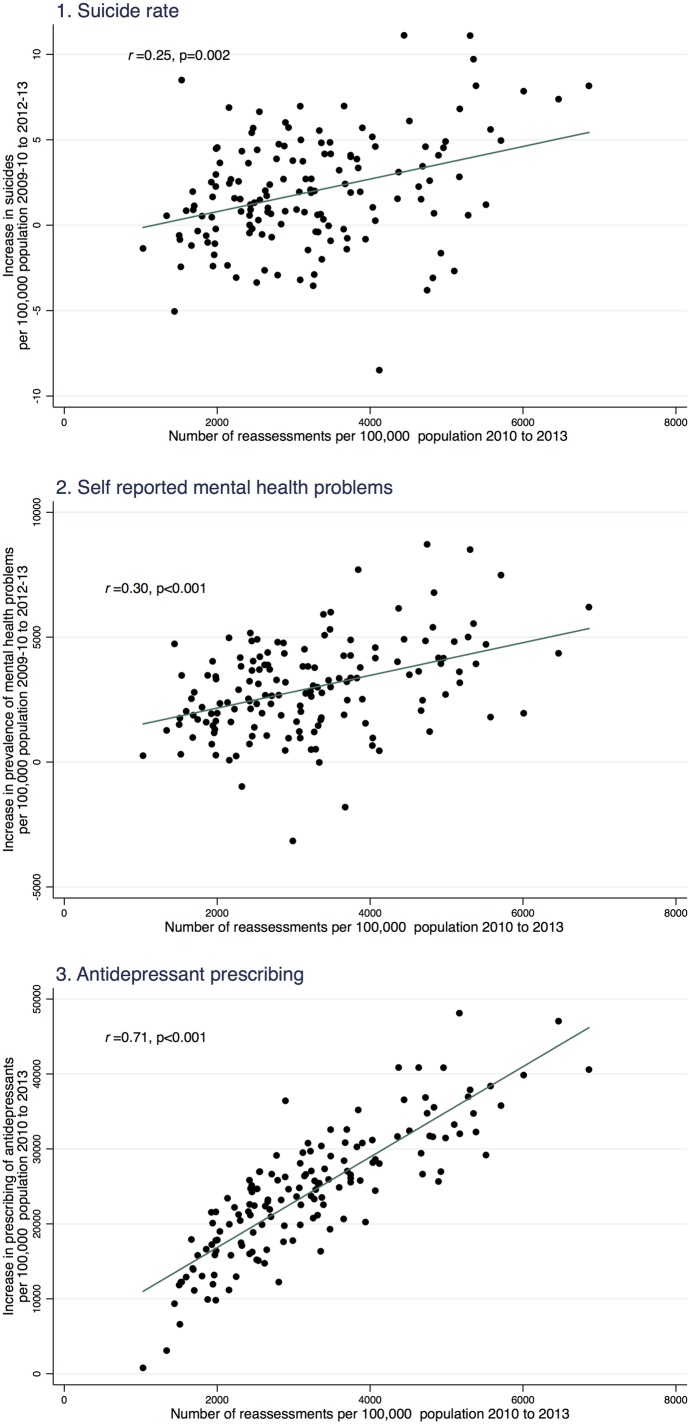
Association between the number of people per 100 000 Working age population experiencing a reassessment in each local authority between 2010 and 2013 and the increase in suicides, self-reported mental health problems and antidepressant items prescribed during the same period, adjusted for area deprivation.

Between 2010 and 2013, 1.03 million existing claimants of out-of-work disability benefits in England were reassessed using the WCA (80% of existing claimants). This is equivalent to 3010 people experiencing a reassessment per 100 000 working age population. The reassessment rate varied across the country from 1030 per 100 000 population in Wokingham (71% of existing clients) to Knowsley where 6860 per 100 000 population experienced a reassessment (88% of existing claimants). As people living in deprived parts of the country are more likely to be receiving disability benefits, a higher proportion of the population in these areas experienced reassessment (see online supplementary appendix 2 for details). Figure 1 shows the association between the proportion of people experiencing reassessment in each local authority between 2010 and 2013 and the change in each of the mental health outcomes between those time periods, adjusted for baseline area deprivation. In those areas where more people had experienced reassessment there was a greater increase in suicides, self-reported mental health problems and antidepressant prescribing.

The multivariable regression analysis indicates that these associations remained after adjusting for other baseline area characteristics, economic trends and long-term trends over time in our three mental health outcomes. The estimates from these models shown in table 1 indicate that for every 10 000 people reassessed there were approximately an additional 6 suicides (95% CI 2 to 9), 2700 cases of reported mental health problems (95% CI 548 to 4840) and 7020 items of antidepressants prescribed (95% CI 3930 to 10 100).

**Table 1:** Additional adverse mental health outcomes associated with each 10 000 people in an area experiencing reassessment

	Number	95% CI		p Value
Suicides	5.68	2.12	9.23	0.002
Cases of mental health problems	2700	548	4840	0.014
Items of antidepressants	7020	3930	10 100	<0.001

Models based on equations shown in online supplementary appendix 3 and included controls for local authority fixed effects, time trends 2004 to 2006 and 2007 to 2013, season, quarterly unemployment rate, annual GVA, annual median wages, annual local authority expenditure and separate time trends by quintile of deprivation and government office region. (Full model results are given in online supplementary appendix 3).

GVA, gross value added.

In total, across England as a whole, the WCA disability reassessment process during this period was associated with an additional 590 suicides (95% CI 220 to 950), 279 000 additional cases of self-reported mental health problems (95% CI 57 000 to 500 000) and the prescribing of an additional 725 000 antidepressant items (95% CI 406 000 to 1 045 000). To put this into perspective of overall levels of these outcomes, this is equivalent to 5% of the total number of suicides, 11% of prevalent cases of self-reported mental health problems and 0.5% of the total number of antidepressant items prescribed in England. As more disadvantaged socioeconomic groups are more likely to be in receipt of disability benefits, and thus to be assessed, the reassessment policy was associated with a greater increase in these adverse mental health outcomes in more deprived areas (see online supplementary appendix 6).

### Robustness tests

We found no significant association between the reassessment rate and trends in self-reported mental health problems and suicides in the over 65-year-old population, (ie, people over retirement age and therefore not subject to the WCA reassessment process). We also found no association with trends in heart conditions in the working age population, or trends in prescribing of cardiovascular drugs. (ie, health conditions that would not plausibly be affected by the WCA reassessment process, in the short term at least). These test results suggest that the observed association between the reassessment process and mental health outcomes in the working-age population is not due to unobserved confounding (see online supplementary appendix 4).

In the lagged analysis, we found that the level of reassessment in the previous time period predicted future increases in suicides, self-reported mental health problems and antidepressant prescribing. The effect sizes were significant and larger than those estimated using the cumulative measure (see online supplementary appendix 4). To further test for reverse causality, we investigated whether the trend in each of the mental health outcomes predicted future increases in the reassessment rate and found no significant association (see online supplementary appendix 4).

As our main analysis was based on aggregate data, it is possible that changes in composition of these populations could explain the results. To explore this further we analysed individual level data from the Labour Force Survey in a multilevel model further controlling for a number of individual characteristics, including age and sex, labour market status (employed, unemployed and inactive), number of physical chronic illnesses and level of education. This analysis gave very similar results as that based on aggregate data (see online supplementary appendix 4).

In additional analysis we also controlled for differential trends by the level of rurality in each area and trends in initial assessments for out-of-work disability benefits and found these did not change our results (see online supplementary appendix 5).

## Conclusion

We found that those local areas where a greater proportion of the population were exposed to the reassessment process experienced a greater increase in three adverse mental health outcomes—suicides, self-reported mental health problems and antidepressant prescribing. These associations were independent of baseline conditions in these areas, including baseline prevalence of benefit receipt, long-term time trends in these outcomes, economic trends and other characteristics associated with risk of mental ill-health. These increases followed—rather than preceded—the reassessment process.

### Strengths and limitations

There are several strengths to our analysis that enhance its validity. First we find consistent results across three separate mental health outcomes, derived from independent data sources, reducing the likelihood that the results are due to spurious associations. Second our estimated effect sizes were large and statistically significant, when controlling for baseline differences between local authority areas, trends in socioeconomic factors associated with mental health and differential trends by level of baseline deprivation. We also found that the lagged reassessment rate predicted future increases in the mental health outcomes, indicating that it is unlikely that the associations that we observed are due to reverse causality.

Some limitations remain, however. As our main analysis was based on aggregate data we cannot identify whether the additional people experiencing the adverse mental health outcomes are the same people who have undergone reassessment. However, we found similar results when we used individual data on mental health problems in a multilevel model to adjust for changes in the composition of local authority populations over time.

It is possible that the association between the reassessment process and adverse mental health outcomes in our analysis was due to unobserved confounding factors. A key assumption is that the variations in local trends in the reassessment rate conditional on the other covariates in our model were not associated with other causes of adverse mental health. As the reassessment process was targeted at more deprived areas and regions, it progressed more rapidly in these areas and a greater proportion of the population was affected (see online supplementary appendix 5). However, we controlled for baseline differences between areas and these differential trends in the analysis. The variation in the reassessment rate that was not explained by the control variables included in our models had no obvious geographical pattern (see online supplementary appendix 5). Reports on the implementation of the reassessment programme indicate that there was geographical variation in the implementation process, due to technical problems, problems with recruiting staff and underestimates of the resources required in some areas to conduct the reassessments.^17^ ^31–34^ It is unlikely that the variation that resulted from these local administrative processes was associated with other causes of adverse mental health. When we replicated the analysis, using outcomes and population groups that should not be influenced by the reassessment process but that could be influenced by unobserved confounding factors, we found that there was no significant association with these outcomes. This adds strength to the conclusion that the association between the reassessment process and adverse mental health outcomes was not due to unobserved confounding.

Patterns of self-reported mental ill-health and antidepressant prescribing may reflect differences in access to healthcare. We adjusted for baseline differences between areas, however, as well as separate regional time trends, which would account for most differences in access. It is unlikely that there would have been sudden increases in access between 2010 and 2013 that would explain recent increases in these measures beyond long-term trends. Analysis of suicides in small areas needs to be interpreted with caution because of the varying use of narrative verdicts by coroners.^35^ However, inclusion of injuries of undetermined cause should have largely dealt with this potential source of bias, and such biases are probably relatively constant over time, making estimates of changes within local authority areas more consistent for testing our study's hypothesis.

### Policy implications

Our results have important implications for policy. The WCA and reassessment policy, was introduced without prior evidence of its potential impact or any plans to evaluate its effects. As pointed out by Petticrew “The public are frequently ‘enrolled’ in real-life policy ‘experiments’ without giving their explicit consent, or indeed without any real prospect of anyone learning anything substantial about the effects of those interventions.” (ref. 36, p.411) Our study provides an initial investigation of the mental health effects of this natural policy experiment, indicating that it may have had substantial adverse consequences for mental health. Health professionals are involved in carrying out a large number of these assessments every year with a further one million assessments planned for 2015.^37^ Given that doctors and other health professional have professional and statutory duties to protect and promote the health of patients and the public,^38^ our evidence that this process is potentially harming the recipients of these assessments raises major ethical issues for those involved. Regulators and other bodies representing health professionals should advocate for the benefits and harms of alternative disability assessment policies to be established though a well-designed trial.

In assessing the costs and benefits of policies that introduce tougher medical assessments for disability benefits, policymakers need to take into account the consequences, not only in terms of the effects on employment, but also the impact on health and the risk of poverty of people with disabilities. Our previous systematic review of international evidence^20^ has indicated that similar policies have tended to shift people from disability benefits to other benefits (eg, unemployment benefits) rather than moving people into employment. Our study provides evidence that the policy in England of reassessing the eligibility of benefit recipients using the WCA may have unintended but serious consequences for population mental health, and there is a danger that these adverse effects outweigh any benefits that may or may not arise from moving people off disability benefits.

As austerity measures designed to reduce public spending increasingly target social protection systems for people with disabilities, the cumulative impact of these developments needs to be assessed.^39^ ^40^ Although the explicit aim of welfare reform in the UK is to reduce ‘dependency’, it is likely that targeting the people living in the most vulnerable conditions with policies that are harmful to health, will further marginalise already excluded groups, reducing, rather than increasing, their independence.

### License

The Corresponding Author has the right to grant on behalf of all authors and does grant on behalf of all authors, a worldwide licence to the Publishers and its licensees in perpetuity, in all forms, formats and media (whether known now or created in the future), to (1) publish, reproduce, distribute, display and store the Contribution, (2) translate the Contribution into other languages, create adaptations, reprints, include within collections and create summaries, extracts and/or, abstracts of the Contribution, (3) create any other derivative work(s) based on the Contribution, (4) to exploit all subsidiary rights in the Contribution, (5) the inclusion of electronic links from the Contribution to third party material where-ever it may be located; and, (6) licence any third party to do any or all of the above.

**What is already known on this subject**

- Since 2010 over a million claimants of the main out-of-work disability benefit in the UK had their eligibility reassessed using a new tougher assessment.
- Doctors and disability groups have raised concerns that this process has had a negative effect on the mental health of the claimants.
- There have not previously been any studies investigating the impact of this or similar policies on mental health.

**What this study adds**

- Those local areas in England where there was a greater increase in the population exposed to the reassessment process experienced a greater increase in three adverse mental health outcomes—suicides, self-reported mental health problems and antidepressant prescribing.
- The reassessment policy may have had serious adverse consequences for mental health in England.
- The health impact of alternative disability assessment policies should be established through well-designed trials before they are implemented universally.

## Supplementary Material

Web supplement
