# Supplementary material for: ‘First, do no harm’: are disability assessments associated with adverse trends in mental health? A longitudinal ecological study
Source: J Epidemiol Community Health. 2015 Nov 16;70(4):339–45. doi: 10.1136/jech-2015-206209 (PMC4819657; doi:10.1136/jech-2015-206209)

Web Appendices. 'First, do no harm': Are disability assessments associated with adverse trends in mental health? A longitudinal ecological study.

Appendix Web 1.

Self reported mental health problems in the Quarterly Labour Force Survey.

In the labour force survey respondents are first asked:

Do you have any health problems or disabilities that you expect will last for more than a year? [LNGLIM]

They are then asked:

Do you have... *Code all that apply* [HEAL0…HEAL09]

1 problems or disabilities (including arthritis or rheumatism) connected with your arms or hands?
2 ...legs or feet?
3 ...back or neck?
4 difficulty in seeing (while wearing spectacles or contact lenses)?
5 difficulty in hearing?
6 a speech impediment?
7 severe disfigurements, skin conditions, allergies?
8 chest or breathing problems, asthma, bronchitis?
9 heart, blood pressure or blood circulation problems?
10 stomach, liver, kidney or digestive problems?
11 Diabetes?
12 depression, bad nerves or anxiety?
13 Epilepsy?
14 severe or specific learning difficulties?
15 mental illness or suffer from phobias, panics or other nervous disorders?
16 progressive illness not included elsewhere (eg cancer not included elsewhere, multiple sclerosis, symptomatic HIV, Parkinson's disease, Muscular Dystrophy)?
17 other health problems or disabilities?

We defined people as having a self-reported mental health problem if they replied yes to the first question [LNGLIM] and were coded as 12 or 15 in any of their responses to the second question [HEAL0…HEAL17]. They were coded as not having a self reported mental health problem if they did not give a response coded as 12 or 15 but did provide a valid answer to the first question.

Discontinuities in the LFS health module.

Between quarter four 2009 and quarter one 2010 the ONS noted that there appeared to be a discontinuity in disability rates calculated from the QLFS. This was not due to any change in the questions and appears to have been due to the addition of a short introduction at the start of the health module:
"*I should now like to ask you a few questions about your health. These questions will help us estimate the number of people in the country who have health problems."*

This resulted in a small increase in the proportion of the population reporting health problems, but there was no change in the characteristics of this population and the ONS concluded that this increase was random. [1] It therefore should not bias results in this analysis.

In 2013 Q2 the filter question identifying people with long term health problems was changed from:

Do you have any health problems or disabilities that you expect will last for more than a year? [LNGLIM]

To

Do you have any physical or mental health conditions or illnesses lasting or expecting to last 12 months or more? [LNGLST]

The questions referring to the types of health problems [HEAL0…HEAL17] remained the same.

To adjust for these changes in the questionnaire we included we included a dummy variable indicating the periods 2010q1 to 2013q1 and 2013q2-2013q4 in the regression model.

Correlation between self reported mental health problems and antidepressant prescribing rates.

We show below that local authority prevalence rates of mental health problems defined in this way correlates reasonably closely with antidepressant prescribing rates, both in terms of level and in terms of change over time.

Figure 1 shows the prevalence in mental health problems for each local authority reported in the labour force survey correlated with the rate of antidepressant prescribing in each area. We also find that the change in the prevalence of mental ill-health reported in an LA is associated with the change in the antidepressant prescribing rate (see Figure 2). As the estimates of the prevalence of mental ill-health are based on quite small samples in each LA, there is some degree of random measurement error, this is exacerbated in the differenced

analysis in Figure 2. However even though there is quite a lot of random noise in the data the fact that we still find a relatively high level of correlation in Figure 2 – indicates that these two indicators are measuring similar phenomena, namely the burden of diagnosed common mental health problems in the population.

Figure Web 1. Correlation between quarterly antidepressant prescribing rate and quarterly prevalence of mental health problem reported in Labour Force Survey for each upper tier local authority in England.

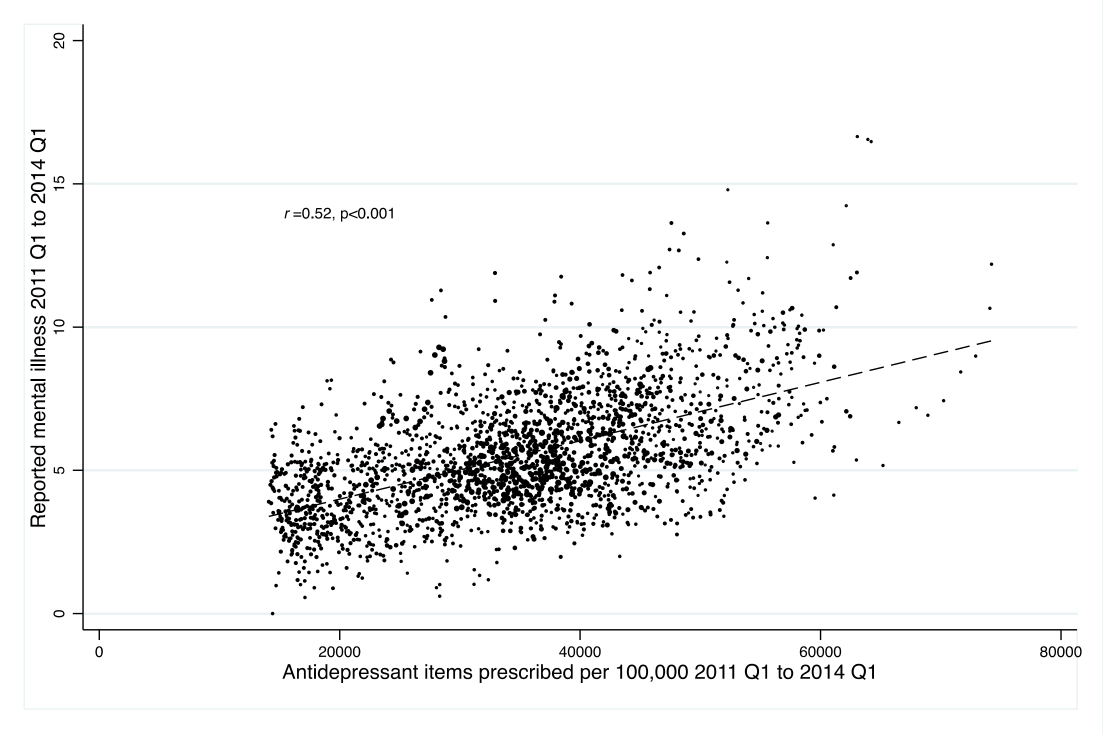

Figure Web 2. Correlation between change over 3 years in antidepressant prescribing rate and quarterly mental health problems prevalence reported in Labour Force Survey within each upper tier local authority in England.

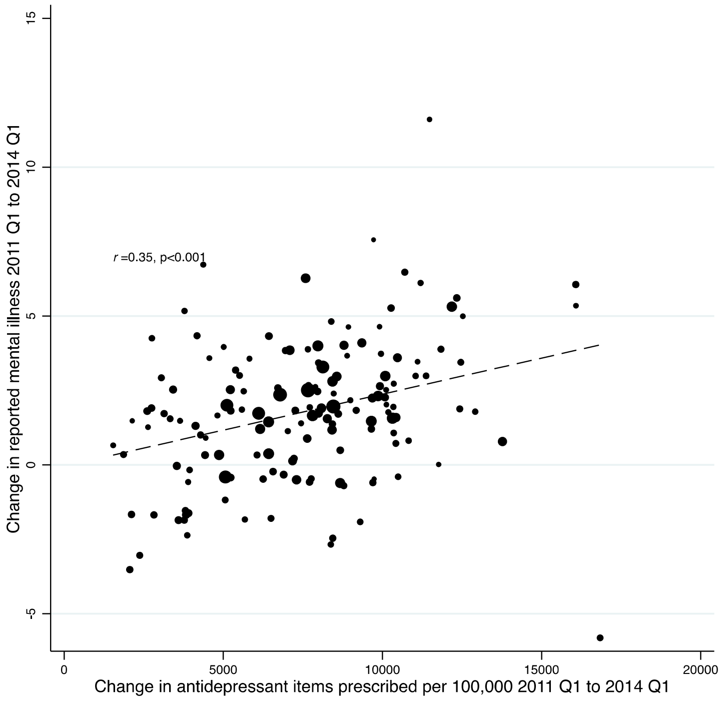

Appendix Web 2. Summary Statistics of exposure (reassessment rate) and outcomes (suicide, self reported mental health problems, and antidepressant prescribing) For England as whole and by level of deprivation 2010 to 2013.

**Reassessments between 2010-2013**					
	**Area**	**Number**	**Average rate per 100,000**	**Min**	**Max**
**Quintiles of deprivation (IMD)**	**Total**	1033600	1,920	646	4401
	**1**	176160	1198	646	1834
	**2**	233170	1654	1185	2252
	**3**	177330	2028	1266	2851
	**4**	219100	2636	1697	3533
	**5**	227840	2786	1516	4401
**Suicides 18-64 year olds between 2010-2013.**					
	**Area**	**Number**	**Average rate per 100,000**	**Min**	**Max**
**Quintiles of deprivation (IMD)**	**Total**	14994	12	5	19
	**1**	3789	11	5	17
	**2**	3713	12	6	15
	**3**	2556	12	8	17
	**4**	2554	13	7	18
	**5**	2382	12	6	19
**Self reported prevalence of mental health problems 18-64 year olds between 2010-2013**					
	**Area**	**Number***	**Average rate per 100,000**	**Min**	**Max**
**Quintiles of deprivation (IMD)**	**Total**	236130	704	356	1237
	**1**	50937	554	384	703
	**2**	54420	609	378	845
	**3**	38440	691	356	1034
	**4**	46613	850	378	1237
	**5**	45720	818	489	1113
**Antidepressant prescribing between 2010-2013.**					
	**Area**	**Number**	**Average rate per 100,000**	**Min**	**Max**
**Quintiles of deprivation (IMD)**	**Total**	167,393,694	300,657	128,327	535,190
	**1**	41692865	274565	158807	342099
	**2**	44958059	307895	152225	439913
	**3**	26455769	290437	138373	444374
	**4**	29849844	343042	128327	505483
	**5**	24437157	286889	136030	535190

***** Estimated from survey data.

Appendix Web 3. Model formula and full model outputs

Model formula.

Specifically we estimated the following model:
Eq 1: $MHOUTCOME_{i,t} = \beta_1 REASSESS_{,I,t} + \beta_2\ UNEMP_{,I,t} + \beta_3\ MEDWAGE_{,I,t} + \beta_4\ GVA_{,I,t} + TIME1 + TIME2 + \beta_5 IMDQ_{I,} x\ TIME1 + \beta_6 GOR_I\ x\ TIME1 + \beta_7 IMDQ_{I,} x\ TIME2 + \beta_7 GOR_I\ x\ TIME2 + CONS + \mu_i + \varepsilon_{i,t}$

Where $MHOUTCOME_{i,t}$ is the mental health outcome in local authority *i* in time t as a rate per 100,000 population.
$REASSESS_{,I,t}$ is the cumulative percentage of the population who have experienced a reassessment in local authority *i* by time t. As the outcome is per 100,000 population this variable is reduced by a factor of 10, so that the coefficient reflects the number of additional cases of the mental health outcome per additional 10,000 people reassessed.
UNEMP is the unemployment rate measured as the proportion of the working age population claiming unemployment benefits in local authority *i i*n time t.
LAEXPRATE is the total expenditure of local authority *i* in year t per head of population in £1000s.
MEDWAGE is the median weekly full time gross wages in £100’s in local authority *i* in time t.
GVA if the Gross Value Added in £1000’s for the region including local authority *i* in time t.
$IMDQ_i$ is the quintile of deprivation of local authority i.
GOR_I is the government office region including local authority i.
μ is a set of local authority dummy variables
TIME1 is a time-trend term. (annual for suicide model and quarterly for self reported mental health problems and antidepressant models)
TIME2 is an additional trend term (spline) to capture any change in trend from 2007.
CONS is a constant.
$\varepsilon_{i,t}$ is an error term

1. Suicide Model

Variable	Coefficient	95% CI	P
REASSESS	5.7	[2.1,9.2]	0.0019
TIME1	-0.2	[-1.5,1.1]	0.746
TIME2	0.2	[-1.3,1.7]	0.8104
UNEMP	0.4	[-0.0,0.8]	0.0529
GVA	0	[-0.1,0.0]	0.3417
MEDWAGE	-0.3	[-1.5,0.8]	0.577
LAEXPRATE	1.1	[-1.0,3.2]	0.3141
1.IMDQ#TIME1	0	[0.0,0.0]	.
2.IMDQ#TIME1	0.2	[-0.5,1.0]	0.5425
3.IMDQ#C.TIME1	-0.7	[-1.7,0.3]	0.1809
4.IMDQ#C.TIME1	0.1	[-0.9,1.0]	0.9063
5.IMDQ#C.TIME1	-0.2	[-1.2,0.8]	0.7006
1.IMDQ#TIME2	0	[0.0,0.0]	.
2.IMDQ#TIME2	0.1	[-1.3,1.5]	0.9309
3.IMDQ#C.TIME2	0	[-1.3,1.3]	0.9991
4.IMDQ#C.TIME2	-1.7	[-3.6,0.2]	0.0756
5.IMDQ#C.TIME2	0.5	[-1.0,1.9]	0.5258
1.GOR#C.TIME1	-0.4	[-1.7,0.9]	0.5863
2.GOR#C.TIME1	0.1	[-1.4,1.6]	0.8652
3.GOR#C.TIME1	-0.4	[-1.9,1.0]	0.543
4.GOR#C.TIME1	-1	[-2.4,0.5]	0.1866
5.GOR#C.TIME1	0	[0.0,0.0]	.
6.GOR#C.TIME1	-0.3	[-1.2,0.5]	0.4711
7.GOR#C.TIME1	0.6	[-0.6,1.8]	0.3391
8.GOR#C.TIME1	-0.5	[-1.6,0.6]	0.3532
9.GOR#C.TIME1	-0.3	[-1.5,0.8]	0.5647
1.GOR#C.TIME2	0	[0.0,0.0]	.
2.GOR#C.TIME2	-0.1	[-1.7,1.5]	0.8942
3.GOR#C.TIME2	0	[-1.4,1.5]	0.9529
4.GOR#C.TIME2	2.2	[0.1,4.3]	0.0394
5.GOR#C.TIME2	-0.4	[-2.0,1.2]	0.644
6.GOR#C.TIME2	0.5	[-1.0,2.0]	0.496
7.GOR#C.TIME2	0.2	[-1.5,1.9]	0.8044
8.GOR#C.TIME2	0.6	[-0.9,2.2]	0.4189
9.GOR#C.TIME2	1.3	[-0.4,2.9]	0.1287
CONS	12.4	[7.2,17.6]	0
N (LA years)	1450		
r2 - within	0.1		

Figure web 3. Checking normality of residuals -- histogram of residuals – Suicide model

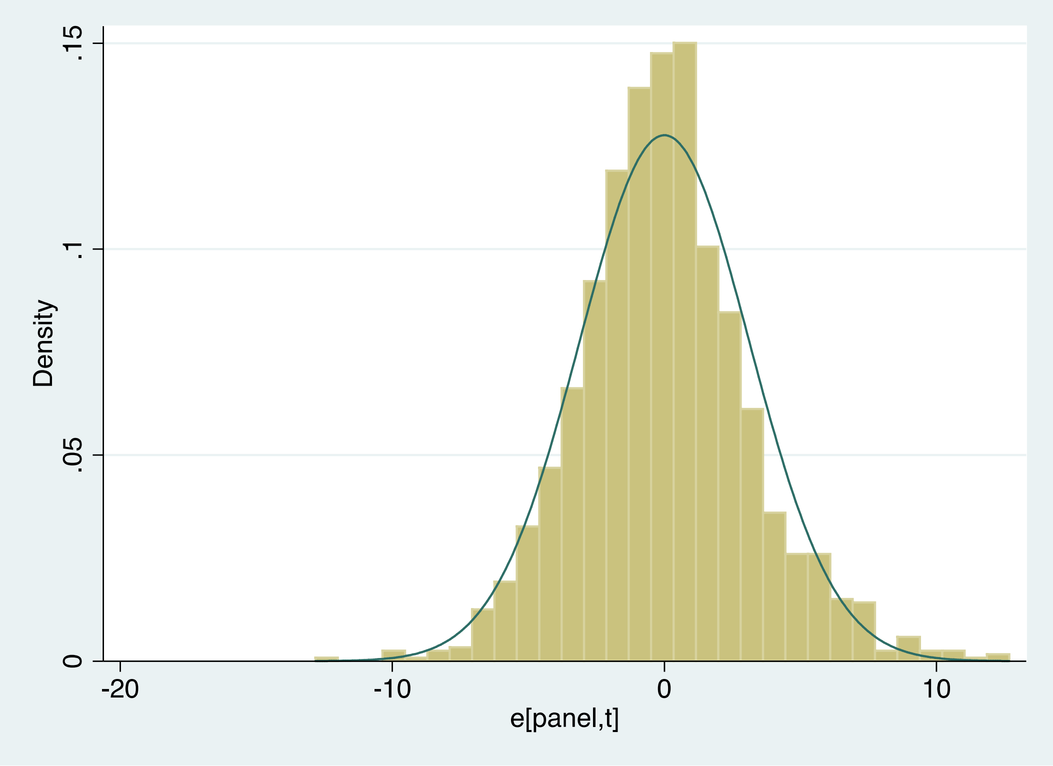

2. Self reported mental health problems.

Variable	Coefficient	95% CI	P
REASSESS	2695.2	[548.0,4842.4]	0.0142
Period 2004q1-2009q4	0	[0.0,0.0]	.
Period 2010q1-2013q1	501.7	[162.1,841.3]	0.0041
Period 2010q2-2013q4	907.8	[346.7,1469.0]	0.0017
TIME1	17.1	[-69.5,103.7]	0.6967
TIME2	58	[-48.4,164.4]	0.2832
Season 1	0	[0.0,0.0]	.
Season 2	109.4	[21.5,197.4]	0.0151
Season 3	74.2	[-26.3,174.8]	0.1466
Season 4	6	[-84.1,96.1]	0.8954
UNEMP	34.4	[-155.1,223.8]	0.7205
GVA	-17.1	[-46.5,12.2]	0.2499
MEDWAGE	-295.7	[-819.6,228.2]	0.2665
LAEXPRATE	191.7	[-952.9,1336.3]	0.7412
1.IMDQ#TIME1	0	[0.0,0.0]	.
2.IMDQ#TIME1	9	[-83.6,101.5]	0.8484
3.IMDQ#C.TIME1	96.3	[-1.5,194.0]	0.0535
4.IMDQ#C.TIME1	38.2	[-113.4,189.8]	0.6194
5.IMDQ#C.TIME1	61.6	[-55.8,179.0]	0.3011
1.IMDQ#TIME2	-2.3	[-114.2,109.5]	0.9676
2.IMDQ#TIME2	-10.2	[-130.3,109.9]	0.8672
3.IMDQ#C.TIME2	-23.3	[-160.9,114.3]	0.7383
4.IMDQ#C.TIME2	-47.3	[-149.5,54.9]	0.3622
5.IMDQ#C.TIME2	0	[0.0,0.0]	.
1.GOR#C.TIME1	7.4	[-82.7,97.5]	0.8713
2.GOR#C.TIME1	-65.3	[-139.6,9.0]	0.0846
3.GOR#C.TIME1	77.2	[9.2,145.1]	0.0263
4.GOR#C.TIME1	-55.2	[-152.6,42.3]	0.2649
5.GOR#C.TIME1	0	[0.0,0.0]	.
6.GOR#C.TIME1	-11.9	[-131.2,107.5]	0.8443
7.GOR#C.TIME1	55.5	[-38.0,148.9]	0.2425
8.GOR#C.TIME1	-94.8	[-186.1,-3.4]	0.0421
9.GOR#C.TIME1	57.6	[-71.2,186.3]	0.3783
1.GOR#C.TIME2	0	[0.0,0.0]	.
2.GOR#C.TIME2	-11.4	[-119.8,96.9]	0.835
3.GOR#C.TIME2	-177.7	[-294.2,-61.2]	0.003
4.GOR#C.TIME2	-60.3	[-238.8,118.2]	0.5055
5.GOR#C.TIME2	-67	[-207.6,73.7]	0.3481
6.GOR#C.TIME2	-8.5	[-145.9,129.0]	0.9033
7.GOR#C.TIME2	15	[-124.0,154.1]	0.8312
8.GOR#C.TIME2	10.3	[-157.1,177.7]	0.9035
8.GOR#C.TIME2	61.1	[-68.9,191.2]	0.3544

CONS	5277.8 [3138.1,7417.5]	0
N (LA quarters)	5777	
r2 - within	0.3	

Figure web 4 . Checking normality of residuals - histogram of residuals – Self reported mental health problems model.

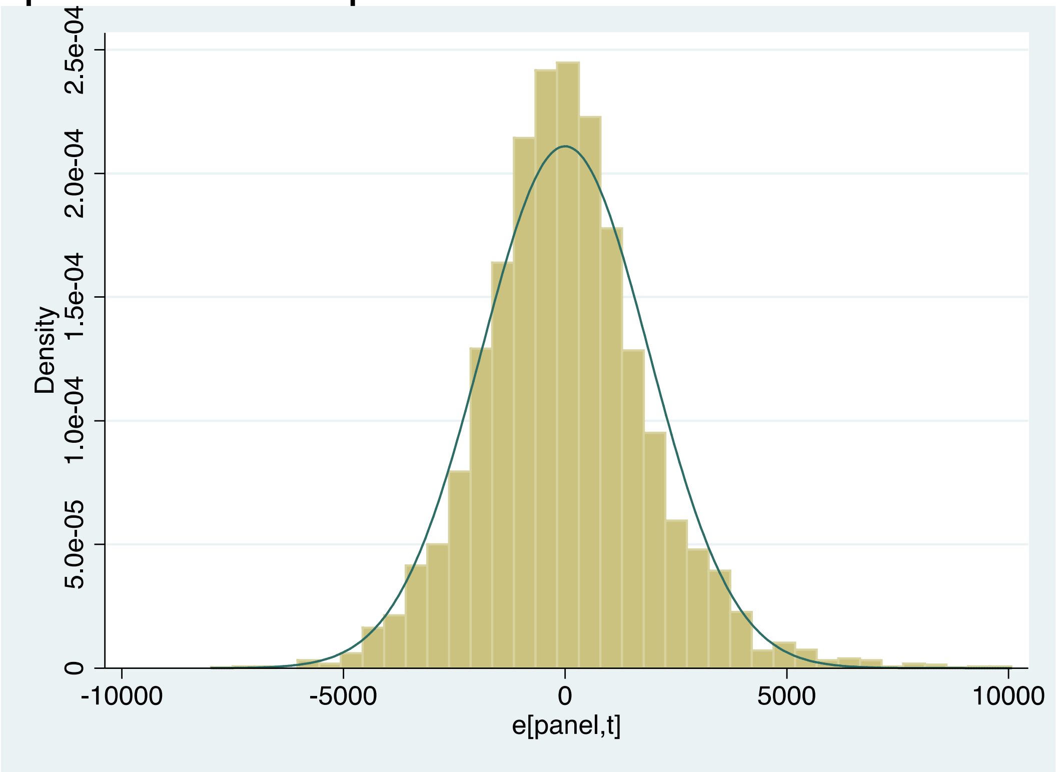

3. **Antidepressant model.**

Variable	Coefficient	95% CI	P
REASSESS	7020.18	[3928.32,10112.05]	0
TIME1	263.21	[194.70,331.72]	0
Season 1	0	[0.00,0.00]	.
Season 2	633.14	[587.50,678.78]	0
Season 3	463.2	[415.96,510.45]	0
Season 4	664.2	[599.14,729.26]	0
UNEMP	379.95	[282.04,477.86]	0
GVA	-3.84	[-57.15,49.47]	0.887
MEDWAGE	-605.36	[-1044.38,-166.34]	0.0072
EXPRATE	1023.25	[97.97,1948.54]	0.0304
1.IMDQ#TIME1	0	[0.00,0.00]	.
2.IMDQ#TIME1	-12.39	[-74.30,49.52]	0.693
3.IMDQ#C.TIME1	-172.83	[-235.70,-109.95]	0
4.IMDQ#C.TIME1	142.86	[54.24,231.48]	0.0018
5.IMDQ#C.TIME1	-10.19	[-73.56,53.17]	0.751
1.GOR#C.TIME1	-70.76	[-131.42,-10.09]	0.0226
2.GOR#C.TIME1	8.71	[-52.40,69.83]	0.7785
3.GOR#C.TIME1	-69.39	[-131.74,-7.03]	0.0294
4.GOR#C.TIME1	3.07	[-65.33,71.47]	0.9295
5.GOR#C.TIME1	0	[0.00,0.00]	.
6.GOR#C.TIME1	57.76	[23.71,91.81]	0.001
7.GOR#C.TIME1	42.78	[-6.37,91.93]	0.0876
8.GOR#C.TIME1	-3.55	[-55.48,48.38]	0.8927
9.GOR#C.TIME1	-15.79	[-79.02,47.44]	0.6225
CONS	8420.76	[4024.54,12816.97]	0.0002
N (LA years)	2086		
r2 - within	0.93		

Figure web 1. Checking normality of residuals -- histogram of residuals – Antidepressant model.

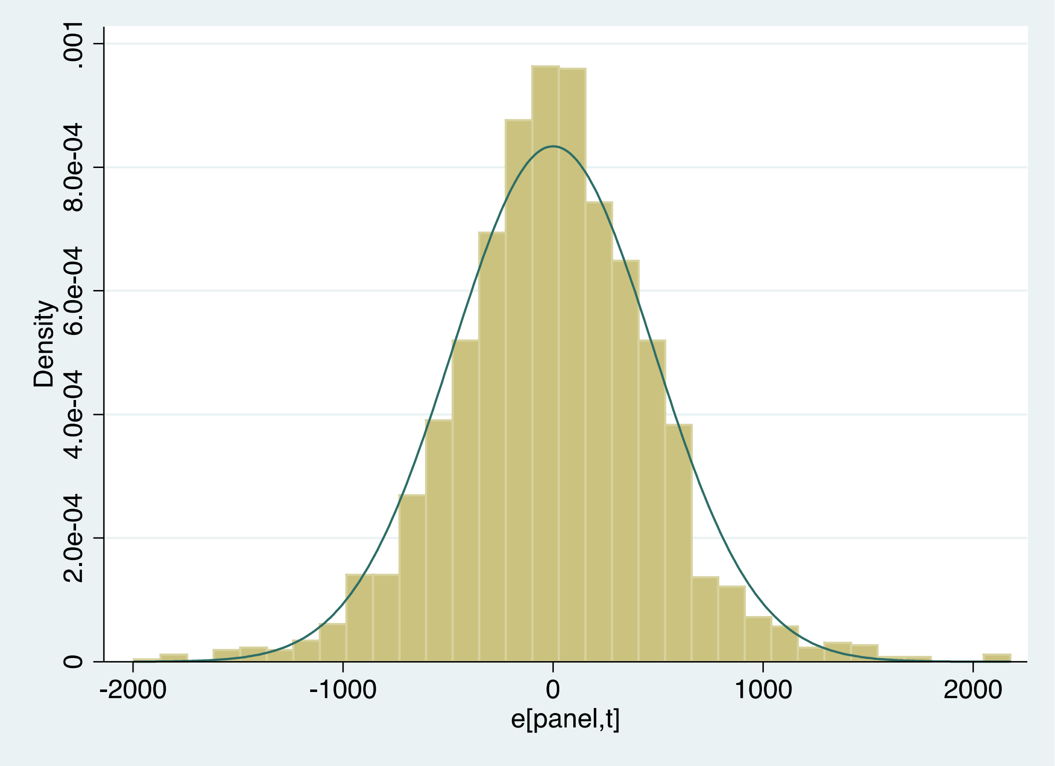

Appendix Web 4. Alternative model specifications.

1. Lagged models.

Table Web 1. Additional adverse mental health outcomes *in current time period* associated with each 10,000 people in an area experiencing reassessment in *the previous time period* (Antidepressants and self reported mental health problems – previous quarter, suicides – previous year).

	Number	95% CI		p
Items of antidepressants	23398	18540	28257	<0.001
Mental health problems	11235	2220	20250	0.01
Suicides	10	3	17	0.01

Note : Models included controls for local authority fixed effects, time trends 2004 to 2006 and 2007 to 2013, season, quarterly unemployment rate, annual GVA, annual median wages, annual local authority expenditure, and separate time trends by quintile of deprivation and government office region.

2. Lead models.

Table Web 2. Additional percentage of the population experiencing reassessment *in current time period* associated with an increase of 1 suicide,1000 cases of self reported mental health problems or 1000 antidepressants prescribed per 100,000 in the previous period *the previous time period* (Antidepressants and self reported mental health problems – previous quarter, suicides – previous year).

	Percentage point increase	95% CI		p
Mental health problems	0.0018	-0.0003	0.004	0.0891
Suicides	-0.0002	-0.0037	0.0033	0.9056
Items of antidepressants	0.0031	-0.0056	0.0118	0.4867

Note : Models included controls for local authority fixed effects, time trends 2004 to 2006 and 2007 to 2013, season, quarterly unemployment rate, annual GVA, annual median wages, annual local authority expenditure, and separate time trends by quintile of deprivation and government office region.

3. Multilevel logistic regression model.

To check whether the association of the reassessment rate with increases in self reported mental il-health, was influenced by changes in the composition of the population we estimated a multilevel model with the reassessment rate at the local authority level along with the quarterly unemployment rate, annual GVA, annual median wages, annual local

authority expenditure, as well as a number of individual level control variables including age and sex, labour market status (employed, unemployed and inactive), number of physical chronic illnesses and socioeconomic group using the National Statistics Socio-economic Classification (NSSEC) groups. The model also included, interactions between sex and age, sex and labour market status and sex and number of physical comorbidities as these had differential effects by gender group.

Table Web 3. Increase in self reported mental health problems associated with each additional 10,000 people in an area experiencing reassessment. – multilevel model.

	Result	95% CI		p
Relative increase - Odds ratio.	1.08	1.06	1.11	<0.001
Absolute marginal increase.	2830	1914	3841	<0.001

Includes controls for local authority fixed effects, time trends 2004 to 2006 and 2007 to 2013, season, quarterly unemployment rate, annual GVA, annual median wages, annual local authority expenditure, and separate time trends by quintile of deprivation and government office region, age and sex, labour market status (employed, unemployed and inactive), number of physical chronic illnesses and educational group.

4. Alternative adjustments for time trends.

In our main model we included data from 2004 in order to take into account trends in our outcomes prior to the implementation of the reassessment process. This is because preexisting trends could act as confounders, for example if trends in suicides were already increasing at a greater rate in areas of the country where the reassessment process proceeded more rapidly this may appear to be the result of the reassessment process if data prior to 2010 was not included. We allow time trends to varying before and after the economic crisis. This is because we know that declining trends in some mental health outcomes such as suicides reversed with the onset of the financial crisis. As there are potentially unobserved confounding factors that had differential trends across regions of the country before and after the recession we allowed underlying trends in mental health outcomes to vary by region and level of area deprivation. In a sensitivity analysis we estimate 3 additional models with simpler time trend structures finding that these tended to result in larger effect sizes, indicating that our preferred model is more conservative and potentially accounts for some unobserved confounders that follow similar time trends.

Model 1. Underlying time trends are assumed not to vary before and after the economic crisis – i.e this model does not include a marginal spline for the 2007-2013 period. i.e

$$MHOUTCOME_{i,t} = \beta_1 REASSESS_{,I,t} + \beta_2\ UNEMP_{,I,t} + \beta_3\ MEDWAGE_{,I,t} + \beta_4\ GVA_{,I,t} + TIME + \beta_5 IMDQ_{I,} x\ TIME + \beta_6 GOR_I\ x\ TIME + \beta_7 IMDQ_{I,} + CONS + \mu_i + \varepsilon_{i,t}$$

Where TIME is a linear trend term, other variable names are as in Appendix 3.

Model 2. Underlying time trends are assumed not to vary before and after the economic crisis AND not to vary across levels of deprivation or regions. i.e

$$MHOUTCOME_{i,t} = \beta_1 REASSESS_{,I,t} + \beta_2\ UNEMP_{,I,t} + \beta_3\ MEDWAGE_{,I,t} + \beta_4\ GVA_{,I,t} + TIME_{,} + CONS + \mu_i + \varepsilon_{i,t}$$

Model 3. The final model was the same as model 2, but was limited to data from 2010 onwards.

The association between the reassessment rate and each of the mental health outcomes estimated from each of these models are given below.

Table Web 4. Additional adverse mental health outcomes associated with each 10,000 people in an area experiencing reassessment estimates with simpler time trend structures (note the antidepressant data was only available from 2010 therefore only model 3 can be estimated)

**Outcome Model**				
**Suicides**	Number	95% CI		p
Model 1	7	4	10	<0.001
Model 2	5	2	7	0.000
Model 3	8	3	14	0.004
**Mental health problems**				
Model 1	3482	1566	5398	<0.001
Model 2	3985	2322	5648	<0.001
Model 3	3808	334	7281	0.032
**Antidepressants**				
Model 3	9291	6882	11700	<0.001

5. Models with alternative groups and outcomes.

To investigate if the association identified in our study was specific to mental health problems in the working age population we repeated the analysis using outcomes we would not expect to be influenced by the reassessment policy. Shadish et al.[2] refer to this as using Nonequivalent Dependent Variables (NDV) i.e those outcomes that should *not* be influenced by a change in the exposure but that could be influenced along with the outcome

by unobserved confounding factors. Finding no effect on these outcomes can enhance the validity of observational analysis.[2] We identified four Nonequivalent Dependent Variables in each of our datasets. Using the Quarterly Labour Force Survey we use the quarterly prevalence of mental health problems in the population over 65 years old and the prevalence of reported Heart, blood pressure & circulation problems in the working age population. Heart, blood pressure & circulation problems were selected as an NDV because it is unlikely that the reassessment process would increase the prevalence of these and this is the largest category of health problems reported in the QLFS. Therefore repeating our analysis with this outcome provides the greatest power to detect any associations. Heart, blood pressure & circulation problems are likely to be affected by other factors that could act as confounders or artifacts in our analysis, such as changes to survey design, changes in the propensity of people to report health problems, changes in access to healthcare, trends in physical health or other confounding factors that are associated with the reassessment rate and trends in this health outcomes. Similarly we investigated whether there was any association between the reassessment rate and trends in the rate of prescribing for cardiovascular conditions (BNF chapter 2). Finally we used data on suicides in over 65 year olds per 100,000 populations as an NDV. This outcome would be sensitive to any changes in the way that suicides are recorded as well as confounding factors that affect suicide risk across all age groups, which could have influenced our results. We find that the reassessment rate is not significantly associated with any of these Nonequivalent Dependent Variables (see Table 5) indicating that it is unlikely that the association that we find between the reassessment rate and trends in adverse mental health outcomes was due to confounding factors or artifacts that would also affect these Nonequivalent Dependent Variables.

Table Web 5. Increase (- decrease) in Nonequivalent Dependent Variables associated with each 10,000 people in an area experiencing reassessment.

	Number	95% CI		p
Items of Cardiovascular drugs prescribed	9644	-4870	24157	0.2
Heart, blood pressure & circulation problems	-1199	-3935	1537	0.4
Mental health problems in over 65 year olds	-1984	-5600	1632	0.3
Suicides in over 65 year olds	3	-2	8	0.2

Note : Models based on equations shown in Appendix 3 and included controls for local authority fixed effects, time trends 2004 to 2006 and 2007 to 2013, season, quarterly unemployment rate, annual GVA, annual median

wages, annual local authority expenditure, and separate time trends by quintile of deprivation and government office region.

Appendix web 5. Investigating variation in reassessment trends.

To make causal inferences about the association between the reassessment rate and trends in adverse mental health outcomes, we need to assume that the variation in local trends in the reassessment rate conditional on other covariates in our model was not associated with other causes of trends in mental health outcomes during this time. In other words we assume that the variation is as good as random. There are a number of reasons that might account for variation in trends in the reassessment rate across local areas. Firstly there is the targeting of the programme at more deprived areas and regions with higher levels of people on Incapacity Benefits, secondly there are logistical, human resource and planning considerations that affect variation in implementation of any large-scale operation. The first of these we control for by including fixed (local authority) effects in the model and separate times trends by area deprivation and region. The remaining variation is therefore likely to be due to these logistical, human resource and planning considerations. We know that there was considerable variation in the implementation process, with some assessment centres progressing at a slower rate than others – leading to a large backlog of claims at some centres. Reports of the reasons for this variation include, technical problems, under estimates of referral rates and the time involved in carrying out assessments when planning resources and problems with recruiting staff [3–7].

To further investigate this variation in reassessment rates we estimate a fixed effects regression model with reassessment trends as the outcome, including the main variables used in the analysis. See table 1. We can see that the reassessment progressed at a faster rate in the North East and North West, in more deprived areas than in more affluent areas, the trend in reassessment was also negatively associated with trends in unemployment, wages and trends in local government expenditure. This indicates that it was necessary to control for these trends in our analysis to reduce possible sources of bias.

Table web 6. Regression model showing association between main control variables and the reassessment rate (people reassessed per 100,000 population).

Variable	Coefficient	95% CI	P
Quarter	108.67	[92.04,125.31]	>0.001
Season 1	0	[0.00,0.00]	.
Season 2	-82.99	[-93.52,-72.46]	>0.001
Season 3	-63.86	[-75.20,-52.52]	>0.001
Season 4	-70.35	[-84.01,-56.69]	>0.001
UNEMP	-347.03	[-390.21,-303.86]	>0.001
GVA	-6.85	[-15.20,1.49]	0.1068
MEDWAGE	-475.53	[-604.75,-346.30]	>0.001
EXPRATE	-1074.8	[-1616.78,-532.83]	0.0001
**Quintiles of deprivation.**			
1.IMDQ#Quarter	0	[0.00,0.00]	.
2.IMDQ#Quarter	30.17	[19.57,40.77]	>0.001
3.IMDQ#Quarter	56.88	[43.98,69.78]	>0.001
4.IMDQ#Quarter	78.96	[64.10,93.83]	>0.001
5.IMDQ#Quarter	104.39	[88.37,120.42]	>0.001
**Regions**			
East Midlands#Quarter	0	[0.00,0.00]	.
East of England#Quarter	-16.63	[-34.22,0.97]	0.0639
London#Quarter	-64.38	[-83.96,-44.81]	>0.001
North East#Quarter	64.47	[20.83,108.11]	0.0041
North West#Quarter	45.27	[26.93,63.61]	>0.001
South East#Quarter	-18.33	[-36.20,-0.46]	0.0444
South West#Quarter	3.77	[-15.54,23.09]	0.7002
West Midlands#Quarter	-17.54	[-38.25,3.17]	0.0964
Yorkshire and the Humber#Quarter	8.25	[-15.80,32.30]	0.4989
CONS	11756	[7860,15651]	<0.001
N (LA years)	2086		
r2	0.84		

We further investigated whether trends in the reassessment rate were additionally associated with trends in initial reassessment rates for Employment Support Allowance in each area and whether the level of rurality in a local authority area influenced the trend in reassessments. It is possible that as the same organisation (ATOS) was carrying out initial assessments during this time high demand of initial assessments in an area may have reduced the rate at which the reassessment programme progressed, it is also possible that

logistical constraints on the programme were greater in more rural areas with more dispersed populations. We divided the local authorities into 5 groups based on the proportion of the population in each LA that was living in a rural area according to Office for National Statistics rural/urban classifications and added interaction terms between level of rurality and time into the model. Regional quarterly caseloads of initial assessments for ESA as a percentage of the working age population were used to assess trends in initial assessment rates. Adding these terms to the model indicated that there was no significant difference in trends in reassessment between more rural or more urban areas, when other covariates were taken into account. However the trend in reassessments was significantly negatively associated with the trend in initial reassessment in an area i.e the reassessment process tended to proceed at a slower rate in areas were there was a greater increase in initial assessments.

Table web 6. Coefficients from regression model showing trends in reassessment rate by level of rurality and association between the reassessment rate and initial assessment rate in each LA . 1.RURAL – least rural quintile to 5.RURAL most rural

**Quintiles of rurality.**	Coefficient	95% CI	P
1.RURAL#Quarter	0	[0.00,0.00]	.
2. RURAL#Quarter	9.51	[-8.64,27.65]	0.3022
3. RURAL#Quarter	-3.98	[-23.31,15.34]	0.6844
4. RURAL#Quarter	14.39	[-9.93,38.71]	0.2443
5. RURAL#Quarter	17.09	[-8.47,42.66]	0.1885
Initial reassessment rate.	-11.65	[-13.74,-9.56]	<0.001

To investigate the geographical pattern of the variation in the reassessment rate that was not explained by our control variables we have mapped the average residuals for each local authority area from the model above (see Figure below). This indicates the variation in the reassessment rate after accounting for the control variables in our model. There is no obvious spatial pattern to this variation, supporting the assumption that it is approximately random.

Figure web 4. Average residuals from model of reassessment rates by local authority area.

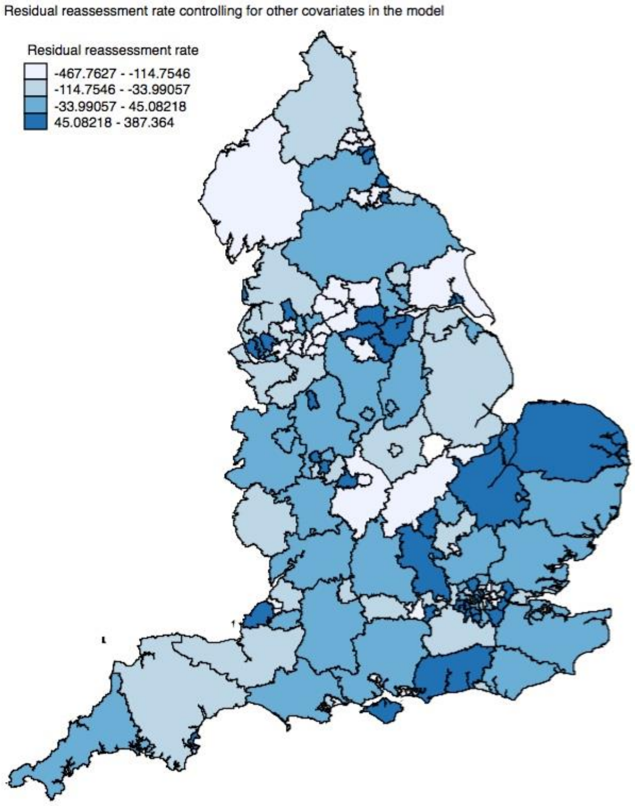

We finally assessed whether including regional trends in initial ESA assessments and separate trends by level of rurality in our model for mental health outcomes affected our results. Local trends in initial assessments for ESA and separate trends by level of rurality were not significantly associated with local trends in any of our mental health outcomes and adding the term to our main models did not change the association between the reassessment rate and the mental health outcomes. (see Table 6 and 7)

Table web 6.Additional adverse mental health outcomes associated with each 10,000 people in an area experiencing reassessment – additionally controlling for trends in initial assessments for ESA and

	Number	95% CI		p
Suicides	6	2	10	0.001
Cases of mental health problems	2270	46	4495	0.045
Items of antidepressants	7002	3898	10106	<0.001

Table web 7.Additional adverse mental health outcomes associated with each 10,000 people in an area experiencing reassessment – additionally controlling for separate trends by level of rurality.

	Number	95% CI		p
Suicides	6	2	9	<0.001
Cases of mental health problems	6708	3762	9653	<0.001
Items of antidepressants	2703	599	4807	<0.001

Appendix web 6. Predicted trends in mental health outcomes in the presence and absence of the reassessment policy by level of area deprivation.

We used out regression models to estimate how the predicted trends of our mental health outcomes would have differed in the absence of the reassessment policy compared to trends in the presence of the policy. To assess the potential impact on health inequalities, we investigated whether the association between the reassessment rate and the mental health outcomes varied by level of baseline deprivation by testing interactions between these variables, and estimated the trends in the most affluent and most deprived parts of the country based on the upper and lower quintiles of area deprivation (IMD). As the relationship between deprivation and antidepressant prescribing is very different within London as compared to areas outside London[1] we presented results for antidepressant prescribing separately for these areas.

Figure 5 shows the estimated trends in each mental health outcome in the most deprived and least deprived areas of England and the predicted trend that would have been expected from the regression models if these 1.03 million people had not been through this reassessment process. There was no significant interaction between the reassessment rate and area deprivation, i.e the same level of increase in the reassessment rate was associated with the same impact in deprived areas as in more affluent areas. However as more disadvantaged socioeconomic groups are more likely to be in receipt of disability benefits, and thus to be assessed, the reassessment policy was associated with a greater increase in these adverse mental health outcomes in more deprived areas. Our analysis shows that the gap in the suicide rate and to a lesser extent self reported mental health problems between the least deprived and most deprived areas had been declining prior to the introduction of the reassessment policy, however after the policy this trend reverses. This suggests that

there would have been a further narrowing of these inequalities in the absence of the reassessment process.

Figure web 5. The estimated trend in suicides, mental health problems and antidepressant prescribing in the most deprived and least deprived local authorities areas in England, dashed lines show the predicted trend in the absence of the reassessment policy, 2004 to 2013.

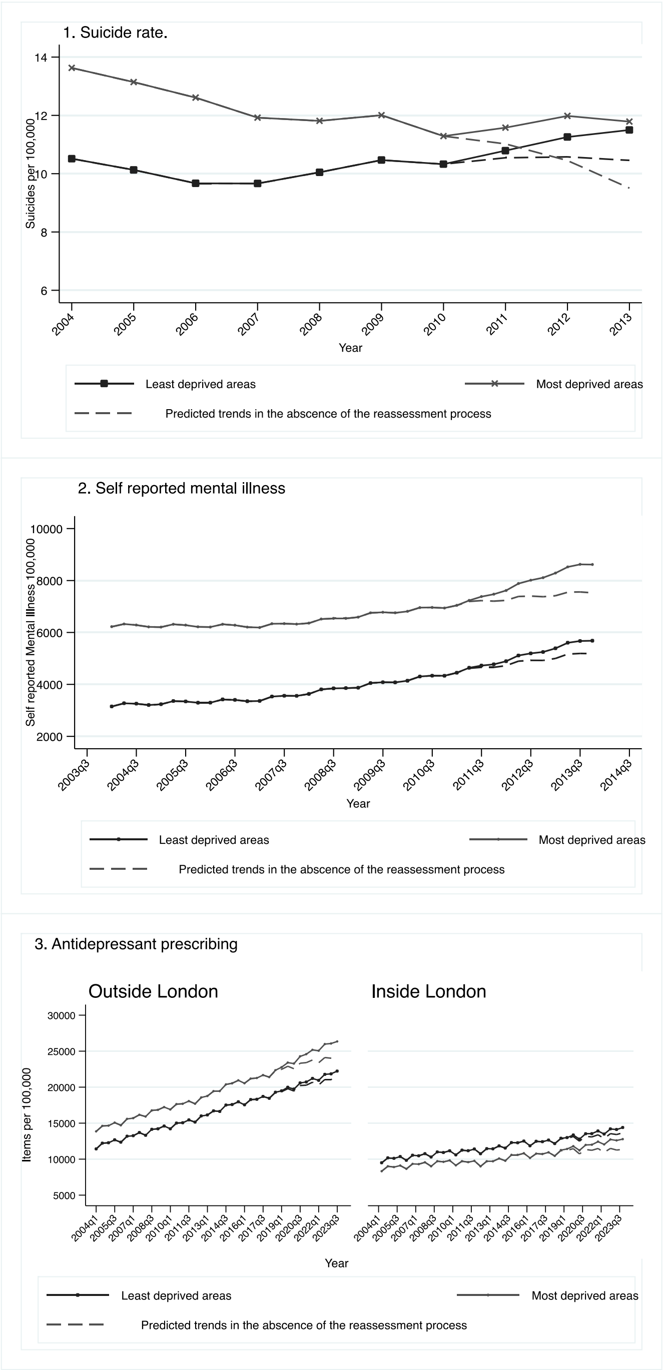

Supplement: Web supplement [file jech-2015-206209-s1.pdf]
